# Triage Knowledge and Practice and Associated Factors Affecting the Accuracy of Nurse Triage in Tertiary Care Among Emergency Departments

**DOI:** 10.7759/cureus.95810

**Published:** 2025-10-31

**Authors:** Reham Mostafa, Khaled El-Atawi

**Affiliations:** 1 Emergency Department, Saudi German Hospital, Dubai, ARE; 2 Pediatrics/Neonatal Intensive Care Unit, Latifa Women and Children Hospital, Dubai, ARE

**Keywords:** accuracy, emergency department, knowledge, nurses, practice, triage, triage decision

## Abstract

Introduction: Emergency essential services employ a triage approach to prioritize patients based on their clinical severity. The objective is to evaluate emergency department (ED) nurses' triage knowledge and practice by identifying factors influencing triage system accuracy. This involves assessing nurses' skills and understanding of triage procedures, potentially through surveys, observations, or performance evaluations, and examining how variables such as experience, training, and hospital type affect triage accuracy, which is essential for efficient and effective emergency care.

Methods: This was a cross-sectional descriptive study conducted from February 2024 to January 2025 on 300 nurses in the ED and 800 patients through a Google Forms questionnaire (Google, Inc., Mountain View, CA), distributed by email at different hospitals in the United Arab Emirates (UAE) and the Kingdom of Saudi Arabia (KSA). Data were gathered using a self-administered questionnaire that employed non-probability purposive sampling to assess sociodemographic data, triage knowledge, and accuracy.

Results: In general, participants exhibited a high knowledge and triage practice level. Nonetheless, it was detected that there was an incorrect practice and a lack of knowledge in relation to certain aspects. According to the demographics of the participants, including qualification, gender, training, job title, and former triage training, there were insignificant differences in triage knowledge and practice (p > 0.05). Between triage knowledge and practice, there was a significant positive relationship (p < 0.01). It was observed that the average duration of accurate triage decisions was longer in patients with triage category 3.

Conclusions: Findings revealed that emergency nurses had moderate levels of triage knowledge. While no statistically significant differences were found based on gender, job title, qualification, or additional training, nurses with more clinical experience showed higher triage accuracy, suggesting that experience may enhance performance.

## Introduction

A critical element of the healthcare delivery system is the emergency department (ED). Healthcare workers in the ED are the first line of defense when it comes to patients who are experiencing acute, life-threatening conditions [1]. The quality of services provided in the ED has substantial implications for the psychological well-being, satisfaction with care, and survival of patients, as well as their hospital experience [2]. The capacity to enhance case flow in hospital units, particularly for required life-saving procedures, is present when the ED operates at its optimal capacity [3].

Triage is a process that involves the timely and precise detection of patients who necessitate instant treatment and the differentiation of those who also present with illnesses or diseases but whose care can be postponed [4]. During pandemics, disasters, and other public health emergencies, effective triage is essential to confirm that the overall patient demand is met by the healthcare organizational capacity [5]. In some cases, however, there are instances of under- and over-triage. Under-triage occurs when patients with rapidly deteriorating clinical conditions are overlooked, while over-triage is the result of prioritizing acute patients with non-life-threatening illnesses, subsequently resulting in a waste of manpower and medical equipment [6].

Every second is crucial for patients in EDs. A patient's death, disability, or recovery from an ED visit can be contingent upon the duration of their stay [7]. The accuracy of triage decisions is another factor that impacts this process [8]. It is the primary responsibility of triage nurses to ensure that patients are in the appropriate location at the appropriate time in the ED and that no one is overlooked during the initial assessment [9]. In EDs, it is estimated that approximately half of the triage assessments are inaccurate [10,11]. Poor clinical outcomes, including prolonged diagnosis and treatment times for patients, improper utilization of hospital resources, reduced patient and employee satisfaction, and even elevated mortality rates, may result from inaccurate or inconsistent triage in EDs [8,10]. Consequently, triage must be expedited and completed within a two- to five-minute timeframe.

In our region, triage practice is a relatively new subject that presents its own set of challenges. Triage error and accuracy rates are not yet known [12]. There is a dearth of research on triage at the national level. Therefore, further research on triage practices is essential. Supervision, quality service delivery, and continuous monitoring will be facilitated by studies that evaluate nurse triage.

We aimed to estimate nurses' practice and knowledge by identifying the variables that impact the accuracy of triage systems in emergency situations.

## Materials and methods

Setting

The data were collected using a Google Forms questionnaire (Google, Inc., Mountain View, CA) that was disseminated via email to various hospitals in the United Arab Emirates (UAE) and the Kingdom of Saudi Arabia (KSA). Level 4 (overcrowded) was identified as the overcrowding emergency score in accordance with the National ED Overcrowding Scale. Nurses with a minimum of one year of emergency experience and six hours of triage training are responsible for performing triage in the ED. The parameter values were in agreement with those identified in prior research that employed the same measure [13].

Study design

This cross-sectional descriptive study was conducted from February 2024 to January 2025 on 800 patients and 300 nurses in the ED. Patient data were collected retrospectively from triage decision records completed by the participating nurses. Accuracy was evaluated for triage categories 1, 2, and 3, based on patient clinical outcomes including intensive care unit (ICU) admission, inter-hospital transfer, or death within 24 hours. These patient data were used solely to assess the accuracy of the nurses' triage decisions. A Google Forms questionnaire was distributed by email to different hospitals in the UAE and KSA. Employing a self-administered questionnaire with non-probability purposive sampling, sociodemographic data, triage knowledge, and accuracy were collected.

Instrumentation

Participant Information Sheet

Each participant's data document includes inquiries regarding ED nurses' sociodemographic characteristics, including age, job title, gender, years of experience as an RN, and educational attainment. Furthermore, it covers queries about the characteristics of professional ED nurses, such as "Have you received training in triage?," "How would you rate the training?," and "How much would you rate your triage knowledge?" [14,15]. On a scale of 1-10, the last two queries were evaluated, with a higher score indicating a higher level of perceived training or triage knowledge. In the present investigation, the scale was administered in the English language. To evaluate its lucidity, legibility, and applicability, the scale was pretested on 30 senior nurses and sent to five experts in the field of triage for face validation. Several modifications were implemented to the layout and formatting features in order to guarantee that the items were easily comprehensible and uncomplicated, as indicated by their responses and comments.

Triage Knowledge and Practices

Utilizing instruments detected by Phukubye et al., triage practices and knowledge were measured [14]. The triage practice and knowledge scale comprises 18 and 14 items, respectively. Each item can be responded to with either "agree" or "disagree". Participants are awarded a score of 1 for correct responses, while a score of 0 is awarded for incorrect responses. The total score for triage practice ranges from 0 to 14, and for triage knowledge, it can range from 0 to 18. A higher total score shows that the triage practice or knowledge is more advanced. Items that are reverse-worded are reverse-coded prior to total score calculation. The instrument now involves six additional multiple-choice questions that pertain to organizational triage system characteristics in the hospital where the emergency department nurses are employed. The measurement's content validity and internal consistency reliability are well-established [14].

Statistical analysis

IBM SPSS version 28 (IBM Corp., Armonk, NY) was employed to conduct statistical analysis. In order to assess nominal organizational triage features, individual socioeconomic factors, and triage practices and knowledge item analysis, descriptive statistics were implemented. Based on sociodemographic characteristics, one-way analysis of variance (ANOVA) and t-tests were implemented to examine variations in triage knowledge and practices. The relationship between triage knowledge and practice was ascertained using the Pearson correlation test. Cronbach's alpha coefficient was employed to ascertain the internal consistency reliability of the triage knowledge and practice scales. Using the Chi-square test, the correlation between categorical variables was investigated. A Bonferroni multiple comparison test was implemented to evaluate group differences subsequent to the Chi-square test. A parametric continuous data set was contrasted using the independent sample t-test.

## Results

Sociodemographic characteristics

Overall, 300 emergency department nurses participated in the investigation. There were 161 nurses who contributed, with a mean age of 30.6 years (standard deviation (SD): 3.41), and 161 (53.7%) of them were female. The following were the percentages of the participants: 235 (78.3%) were registered nurses, 131 (43.7%) had a bachelor's degree in nursing, 250 (83.3%) had former triage training, and 144 (48%) had in-service triage courses, as per the data (Table 1).

**Table 1:** Distribution of sociodemographic characteristics of emergency department nurses (N = 300) (including age, gender, job title, education level, and training history) Data are presented as mean ± SD or number (%). SD: standard deviation

Parameters	Mean ± SD/number (%)
Age (years)	30.6 ± 3.41
Gender	
Male	139 (46.3%)
Female	161 (53.7%)
Job title	
Registered nurse	235 (78.3%)
Nurse specialty	65 (21.7%)
Educational attainment	
Diploma	35 (11.7%)
Bachelor's degree	131 (43.7%)
Postgraduate diploma	66 (22%)
Postgraduate degree	68 (22.7)
Prior triage training	
Yes	250 (83.3%)
No	50 (16.7%)
Additional emergency nursing training or courses	
None	51 (17%)
In-service course in triage	144 (48%)
Emergency nursing certificate	90 (30%)
Others	15 (5%)

Triage knowledge

The statements with the highest level of agreement among the participants were as follows: triage involves categorization of patients according to the priority of their injuries or illness (288 (96%)), the purpose of triage is to prevent patients from deteriorating or dying (289 (96.3%)), patients with higher social status are managed more promptly even if they are triaged as green (237 (79%)), and triage knowledge is not significant (288 (96%)).

Utilizing a scale ranging from 1 to 10, with 10 demonstrating the highest level of knowledge, contributors were asked to rate their level of triage knowledge. It is important to note that 75.7% of the contributors agreed with item number 10, which is connected to AVPU (alert, verbal, pain, and unresponsive). This is incorrect because the letter "p" is associated with pain, not pulse. The self-rating score and the rating that applicants assigned to the caliber of their ED triage instruction were comparable. The average score for triage knowledge was 13.91 (SD: 3.16) (Table 2).

**Table 2:** Distribution of the responses to triage knowledge items by emergency department nurses (with percentages) Data are presented as mean ± SD or number (%). SD: standard deviation

Statement	Agree	Disagree
1. Triage is the sorting of patients into priority of injuries or illnesses.	288 (96%)	12 (4%)
2. The purpose of triage is to prevent deterioration or death of a patient while waiting in the queue for their turn.	289 (96.3%)	11 (3.7%)
3. Triage early warning signs is short (triage early warning signs).	250 (83.3%)	50 (16.7%)
4. There are two versions of the Canadian Triage and Acuity Scale (one for children and one for adults).	226 (75.3%)	74 (24.7%)
5. If an emergency sign is identiﬁed in the ﬁrst step, the patient's vital signs are taken ﬁrst.	198 (66%)	102 (34%)
6. If no emergency signs are identiﬁed in step 1 (but an urgent sign is identiﬁed in step 2), the patient is immediately triaged yellow and asked to wait.	180 (60%)	120 (40%)
7. Patients who are Canadian Triage and Acuity Scale priority level yellow should be referred to the designated area for non-urgent cases.	266 (88.7%)	34 (11.3%)
8. Patients triaged as white should wait for 10 minutes before being attended.	147 (49%)	153 (51%)
9. Nursing auxiliaries are not allowed to triage.	184 (61.3%)	116 (38.7%)
10. AVPU is short for alert, verbal, pulse, and unresponsive.	227 (75.7%)	73 (24.3%)
11. Adult Triage Early Warning Score consists of the following parameters: mobility, respiratory rate, heart rate, diastolic blood pressure, temperature, and AVPU.	216 (72%)	84 (28%)
12. A tiny baby under two months should always be referred to the senior healthcare practitioner once they have been comprehensively triaged.	244 (81.3%)	56 (18.7%)
13. Patients with a green triage color (priority 4) should be attended ﬁrst when triaging.	62 (20.7%)	238 (79.3%)
14. The Canadian Triage and Acuity Scale has five color coding or priorities.	262 (87.3%)	38 (12.7%)
15. Triage is difﬁcult and costly to implement in district emergency units.	55 (18.3%)	245 (81.7%)
16. Patients with high social status, e.g., town mayor, school principals, and politicians, should be treated as very urgent even if triaged as color green.	63 (21%)	237 (79%)
17. Discriminator list is not important for triage purposes.	182 (60.7%)	118 (39.3%)
18. Triage knowledge is not important.	12 (4%)	288 (96%)
Overall knowledge score	13.91 ± 3.16	

Triage practice

The average triage practice score was 10.57 (SD: 2.84). Statements 11 and 13 received the greatest agreement from the participants. Statement 11 states that waiting time delays can have a negative impact on patient outcomes (228 (76%)), while statement 13 specifies that shorter waiting times led to fewer crowding and better patient satisfaction (245 (81.7%)). Participants disagreed most with statements 4 and 12, which claim that waiting times in rural regions could never be reduced due to a lack of manpower (87 (29%)) and that the triage primary warning score is calculated after the triage code is assigned (48 (16%)), respectively. Notably, 77.7% of the contributors agreed with item 10, which states that a patient triaged as green should wait for one hour or less. This is incorrect, as the recommended waiting time is two hours (Table 3).

**Table 3:** Distribution of the responses to triage practice statements by emergency department nurses Data are presented as mean ± SD or number (%). SD: standard deviation

Statement	Agree	Disagree
1. The triage process should be practiced by professional nurses only.	282 (94%)	18 (6%)
2. The triage process starts with taking the vital signs of the patient.	280 (93.3%)	20 (6.7%)
3. Allocating a triage code is the last step in the triage process.	237 (79%)	63 (21%)
4. Calculation of triage early warning signs is done after allocating a triage code.	87 (29%)	213 (71%)
5. Comparison of the discriminator list and triage early warning signs score is done before allocating a triage.	188 (62.7%)	112 (37.3%)
6. Triage reduces the waiting time of patients in emergency units.	173 (57.7%)	127 (42.3%)
7. Waiting time should not be considered when rendering emergency care.	253 (84.3%)	47 (15.7%)
8. Waiting time is one of the six ministerial priorities in Saudi Arabia.	146 (48.7%)	154 (51.3%)
9. Patients triaged as yellow should wait for 10 minutes to be seen.	173 (57.7%)	127 (42.3%)
10. Patients triaged as green should wait for one hour or less.	233 (77.7%)	67 (22.3%)
11. Delays in waiting time can negatively impact the outcomes of the patient's condition.	228 (76%)	72 (24%)
12. Waiting time can never be improved in a rural hospital due to a shortage of human resources.	48 (16%)	252 (84%)
13. Short waiting time in emergency units reduces overcrowding and results in patient satisfaction.	245 (81.7%)	55 (18.3%)
14. It is illegal to delay triage in patients within emergency units.	267 (89%)	33 (11%)
Overall practice score	10.57 ± 2.84	

Comparisons between knowledge and practice

Triage knowledge and practice did not differ significantly from prior triage training, qualification, job title, gender, or further nurse training in emergency taken (p > 0.05) (Table 4).

**Table 4:** Correlation between triage knowledge and practice based on sociodemographic variables r: Pearson coefficient *Statistically significant at p ≤ 0.05

Parameters	Triage knowledge		Triage practice	
	r	p-value	r	p-value
Gender	-0.029	0.621 (>0.05)	-0.029	0.618 (>0.05)
Job title	-0.080	0.168 (>0.05)	-0.068	0.240 (>0.05)
Educational attainment	0.004	0.384 (>0.05)	-0.007	0.328 (>0.05)
Prior triage training	0.098	0.091 (>0.05)	0.084	0.062 (>0.05)
Additional emergency nursing training or courses	0.035	0.389 (>0.05)	-0.041	0.136 (>0.05)

Relationship between triage practice and knowledge

Across triage practice and knowledge, a significant positive moderate relationship was observed (p < 0.001, r = 0.951), suggesting that better triage practices are linked to greater triage knowledge (Table 5).

**Table 5:** Correlation between triage knowledge and practice scores r: Pearson coefficient *Statistically significant at p ≤ 0.05

Variables	r	p
Knowledge versus practice	0.951	<0.001*

Nurse triage decision accuracy and time duration

In cases with triage category 2, no difference was observed between precision decision and triage times. While in category 1, the accurate triage decision average time (p = -) denotes an inaccurate triage decision, although without significance. Furthermore, patients in triage category 3 had longer triage times based on valid triage decisions (p = 0.022). The average time and accuracy of choices varied between patients in triage categories 4 and 5. Category 4 (p < 0.001) and category 5 (p = 0.002) had lower average times for valid triage choices (Table 6).

**Table 6:** Relation between triage decision accuracy and time duration across categories 1-5 Data are presented as mean ± SD or number (%). SD: standard deviation, t: Student t-test, p: p-value for the association between inaccurate and accurate *Statistically significant at p ≤ 0.05

Triage category	Triage decision	Triage duration (seconds)		t-test	p
		Number (%)	Mean ± SD		
1	Inaccurate	4 (1%)	1.25 ± 0.29	-	-
	Accurate	-	-		
2	Inaccurate	45 (11.6%)	1.88 ± 1.30	0.366	0.715 (>0.05)
	Accurate	54 (13.1%)	1.78 ± 1.20		
3	Inaccurate	186 (47.8%)	1.73 ± 1.15	2.294	0.022* (≤0.05)
	Accurate	203 (49.4%)	2.02 ± 1.34		
4	Inaccurate	149 (38.3%)	1.75 ± 1.20	11.664	>0.001*
	Accurate	149 (36.3%)	0.60 ± 0.0		
5	Inaccurate	5 (1.3%)	2.76 ± 0.69	6.957	0.002* (≤0.05)
	Accurate	5 (1.2%)	0.60 ± 0.0		

Triage accuracy by shift

Nurse triage accuracy rates by shift differed statistically significantly. The higher case density during the shift in the evening compared to the shifts in the day and night (p = 0.030) explains this difference, according to the Bonferroni test (Table 7).

**Table 7:** Relation between shift and triage decision Data are presented as number (%). c2: Chi-square test, p: p-value for association between shift categories *Statistically significant at p ≤ 0.05

Triage decision	Shift			c^2^	p
	Day (08:00-15:59) (n = 294)	Evening (16:00-23:59) (n = 438)	Night (00:00-07:59) (n = 68)		
	Number (%)	Number (%)	Number (%)		
Inaccurate	135 (45.9%)	229 (52.3%)	25 (36.8%)	7.037	0.030* (≤0.05)
Accurate	159 (54.1%)	209 (47.7%)	43 (63.2%)		

The accuracy rates of nurse triage were statistically significantly different based on the working duration of the nurses in triage. Specialist nurses have a higher triage accuracy rate (126 (56.5%)) than neophyte nurses (14 (28.6%)), as indicated by the Bonferroni test (p = 0.012). The triage accuracy rates of novice nurses are low (Table 8).

**Table 8:** Relation between decision working time in triage (months) and triage decision Data are presented as number (%). c2: Chi-square test, p: p-value for the association between decision working time in triage (months) categories *Statistically significant at p ≤ 0.05

Triage decision	Decision working time in triage (months)					c^2^	p
	0-11 months (novice) (n = 49)	12-23 months (experienced) (n = 10)	24-35 months (proficient) (n = 153)	36-59 months (master) (n = 365)	60 months and ↑ (expert) (n = 223)		
	Number (%)	Number (%)	Number (%)	Number (%)	Number (%)		
Inaccurate	35 (71.4%)	5 (50%)	72 (47.1%)	180 (49.3%)	97 (43.5%)	12.774	0.012* (≤0.05)
Accurate	14 (28.6%)	5 (50%)	81 (52.9%)	185 (50.7%)	126 (56.5%)		

## Discussion

Triage is a component of contemporary ED care. Patients' needs are prioritized by nurses in severe illness during triage; they must be trained and experienced appropriately to enhance their triage abilities [16].

In his study, Elgazzar found that nearly 50% of nurses had a moderate level of triage knowledge, with a mean score of 10.94 and an overall mean score of 10.94 (SD: 2.18, range: 0-18) [17]. Participants in the current study showed strong agreement with statements describing triage as the process of sorting patients according to the priority of their injuries or illnesses (288 (96%)). The findings showed that the participants' self-rated knowledge level and their measured ED triage knowledge were constant, which may be due to the emergency nurses' greater experience. Reduced ED overloading, shorter wait times, better case movement through the ED, and higher patient satisfaction are all potential high-level outcomes of triage knowledge and proficiency.

Consistent with the current study, Fathoni et al.'s assessment of triage knowledge and skills among emergency center nurses in Dar es Salaam, Tanzania, found that 67% of respondents were knowledgeable about triage, while 33% were not [18]. Moreover, Afaya et al. [19] and Aloyce et al. [20] discovered that a significant number of nurses were informed about triage, which is consistent with the results of the present study. Furthermore, Asgari et al. found that nurses possessed only a limited understanding of triage [21]. In agreement with Fathoni et al., the nurses demonstrated a moderate level of triage abilities [22]. Nurses demonstrated high accuracy in responding to questions about the definition and purpose of triage, who is responsible for triage, initial evaluation, the color coding system, quick estimation, and evaluation at regular intervals, as indicated by the conclusions of the findings.

The investigation by AlShatarat et al. revealed a knowledge deficiency in certain knowledge-related measures (e.g., 68.7% of the participants incorrectly responded to the AVPU) and practice-related items (87.1% of the participants incorrectly recognized the waiting period for patient triage as green) [23]. This implies that to enhance their understanding and triage application, in emergency nursing, the research applicants require advanced education and training. Items relating to the triage description and the critical function that triage plays in avoiding patient mortality and additional disability had the highest agreement records on triage knowledge [3]. The participants' high agreement scores reflected their strong overall levels of triage knowledge. Furthermore, it emphasized that the accurate triage description and the understanding of its advantages for cases arriving at the ED form the basis of every triage nurse's expertise [24]. In addition, the statements suggesting that triage knowledge is not significant and treatment establishment should be grounded on social standing had the greatest disagreement ratings. These findings demonstrated that participants challenged the long-standing practice of ED nurses to prioritize people with high social status, realizing that these decisions ought to end because they can lead to ethical dilemmas when resources and supplies are distributed to people who are not clinically ill but are simply expected to receive special treatment because they are VIPs or very important people [25].

The triage practice that generated the highest agreement scores was the acknowledgment that patients' health can be adversely affected by extended waiting times, while shortened waiting periods can lead to higher satisfaction among patients and reduced overcrowding. These results illustrated that the applicants in their practice made every effort to promptly and accurately triage patients who arrived at the ED and to ensure that they were seen within the standard waiting time. Participants also showed the lowest levels of agreement with the claims that rural triage can never be improved due to staff shortages and that triage early warning scores are incorrectly calculated. Participants acknowledged that the accurate use of early warning scores can enable doctors to make earlier diagnoses and initiate therapy, as well as predict when patients will have bouts of clinical deterioration. Additionally, despite the paucity of manpower, participants acknowledged the significance of enhancing rural ED triage.

According to AlShatarat et al., multinational ED nurses generally had higher levels of ED triage practice and knowledge [23], and their findings were comparatively superior to those reported in previous studies [14,26-28]. The self-rated level of knowledge by the participants and the measured ED triage knowledge were in agreement. Improved patient movement within the ED, reduced ED overcrowding, shorter wait times, and increased case satisfaction could all result from high triage practice and knowledge levels [2]. The participants' levels of triage knowledge and practice can be attributed to the hospital's routine implementation and consistent use of the Canadian Triage and Acuity Scale. Studies focusing on other protocols, such as the Manchester Triage System, have also reported higher triage abilities among emergency nurses, which were attributed to their clinical background [29,30].

Numerous factors, such as the triage nurse's training, years of experience, and certifications, affect their level of knowledge. These factors affect hospital performance, mood, and communication, as well as nurses' involvement in various trainings pertaining to triage judgments. Their comprehensive understanding is demonstrated by their completion of ECG resuscitation training and basic life support [15,31]. The study's findings are comparable to those of prior research and literature that have emphasized the importance of expertise and experience in decision-making during the triage process. The study by Andersson et al. demonstrated that experience is one of the most critical and influential factors in nurses' decision-making during the triage process [32]. The American study by Hicks et al. also noted that greater experience improves the stability of decision-making in triage situations [33]. Additionally, the ED nurses' degree of knowledge at King Fahd Hospital was over 70%, whereas the ED nurses in Dammam Medical Complex in Saudi Arabia had less than 70%, which is regarded as a moderate score. Nurses' inadequate triage knowledge is a result of inadequate educational and training opportunities, and nursing curricula across various programs do not teach the triage system adequately to nurses in emergency units [34]. By giving triage nurses enough time to complete the audit and giving them relevant and helpful feedback, EDs and organizations can encourage auditing.

Moreover, among nurses, higher levels of triage practice and knowledge may be attributed to the successful enforcement of organizational guidelines that ensure ED nurses remain up to date with the latest triage-related knowledge and competencies. Additionally, the implementation of continuous quality improvement initiatives, such as audits, performance evaluations, and regular assessments, can further contribute to enhancing nurses' triage knowledge and practices. In their respective workplaces, an effective and consistent triage system also likely supports and reinforces their understanding and execution of appropriate triage procedures.

In the current study, it was found that although no significant associations were observed between sociodemographic characteristics and triage knowledge or practice, nurses with more years of triage experience exhibited greater accuracy, indicating the potential role of practical experience in refining triage skills. This nuance suggests that while formal training alone may not result in significant differences, accumulated experience enhances decision-making capabilities, especially under high-pressure scenarios typical in emergency departments. This implies that the cohort was homogeneous in terms of triage knowledge and practice, irrespective of their sociodemographic characteristics. This was in stark contrast to the findings of other studies, which revealed substantial correlations between triage knowledge and job title [14], as well as between years of triage knowledge and experience [19], triage knowledge and educational level [26], and work history and triage knowledge in nursing [35].

Elgazzar identified a positive association between nurses' triage knowledge and their years of experience in EDs, noting that triage knowledge improved with extended service in the ED. This enhancement may be attributed to increased clinical exposure, participation in multiple workshops and in-service training sessions, and access to online triage resources [17]. Similarly, Forsgren et al. emphasized that ongoing triage training could significantly enhance nurses' competence in managing complex workplace scenarios [36]. Consistent with previous findings, job experience was shown to correlate with triage knowledge, particularly among experienced nurses over five years, who demonstrated more advanced triage skills compared to their less experienced counterparts [18,32,37].

The relationship between triage practice and knowledge has been the subject of only a few studies. Existing literature demonstrated that triage knowledge predicts triage skills in practice [15]. AlShatarat et al. demonstrated a positive correlation between triage practice and triage knowledge. This suggests that triage practice could be improved by providing additional education on triage knowledge for emergency nurses. Future research should investigate the impact of emergency nursing education programs on the practice of triage among ED nurses in Saudi Arabia [23].

In triage category 3, individuals exhibited a longer average duration for making correct triage decisions. The implication of this outcome is that triage examination durations should be increased, especially for category 3 patients, and should be implemented. In one study, it was determined that roughly half of the patients (49.1%) who applied to the ED were classified as category 3 [6]. According to an additional investigation, nurse triage demonstrated an accuracy rate of 68.3% [38]. The average accuracy rate of nurse triage decisions was 59.2%, and the accuracy rates of these decisions did not differ between regions in another multicenter study [4]. In a study by Chen et al., nurses' triage determinations were 40% inaccurate [38]. Based on a study that evaluated the precision rates of triage decisions using case circumstances, 40.4% of triage decisions were imprecise [39].

The average triage evaluation time in our study was 1.41 ± 1.06 minutes, which is shorter than the recommended timeframe reported in the literature. Furthermore, it was established that the triage duration and the nurse triage decision accuracy rates diverged in categories 3, 4, and 5. In one study, the mean time to complete triage assessment for patients was four minutes and four seconds [40]. In another study, the average triage durations were 2.6 ± 2.5 minutes [41] and 5.9 minutes [42]. In a separate investigation, it was determined that category 3 patients have prolonged triage durations (2.8 ± 2.5 minutes) [41]. It indicates that a triage assessment should be conducted within a two- to five-minute timeframe to ensure that it is both accurate and rapidly conducted.

The number of patients in the ED is one of the most critical variables that influences triage decision-making [43]. The mistake rate was higher in the evening shift with a greater patient density than in the night shift with a less patient density in our study. Triage rate accuracy decreases during evening and day schedules with an increased number of cases. A literature review suggests that nurse triage precision is correlated with the number of working nurses in each shift, the number of patients [44], and workload [10].

As determined by our research, the accuracy rates of nurses increased as their years of triage practice increased. Previous studies found that the accuracy of triage decisions increased with nurses' experience in the ED and their triage practice [38,39]. The accuracy rate of nurses with 3-4 years of triage experience was 67.2%, while those with 0-1 years of triage experience had an accuracy rate of 58.1%, according to the same study [39]. The triage accuracy rate of nurses with less experience was 45.76%, while that of those with more experience was 53.8%, according to a study that investigated the correlation between experience and triage performance [45]. According to Hammad et al., nurses who work in triage should have at least five years of experience [46].

These results indicate that novice nurses should not be employed during daytime and evening schedules in areas with a high patient density. It can be advisable for trainee nurses to collaborate with qualified ones throughout periods of high patient crowdedness to develop their skills and competencies. It also demonstrates the necessity of increasing the number of triage nurses to facilitate the distribution of workload during long shifts.

Limitations

The sample size was relatively small, the study was conducted in a single center, and the follow-up period was relatively short.

## Conclusions

In this study, emergency nurses demonstrated high levels of triage knowledge and practice. Despite that, the study results revealed the existence of some knowledge gaps and deficiencies in triage practice among the study respondents. Accurate triage is vital in emergency departments. However, patient density and time pressure make triage difficult. The findings of this study showed that nurse triage was accurate. The accuracy of nurse triage is affected by seniority, shift, and triage times. This suggests that the study participants still need further education and training in emergency nursing to improve their triage knowledge and practice.

Consequently, the investigation suggests that additional training in emergency nursing should be implemented to rectify knowledge deficiencies and inaccurate triage procedures. In an effort to enhance the precision of nurse triage in the ED, it is imperative to augment the quantity of experienced and well-trained triage nurses, increase the number of nurses in schedules with a high patient volume, and create computer-based guiding support systems. Additionally, there is a requirement for national and international research on the durations of triage assessments and the accuracy/error rates of nurse triage.

## References

[1] Rayan A, Hussni Al-Ghabeesh S, Qarallah I (2022). Critical care nurses’ attitudes, roles, and barriers regarding breaking bad news. SAGE Open Nurs.

[2] Morley C, Unwin M, Peterson GM, Stankovich J, Kinsman L (2018). Emergency department crowding: a systematic review of causes, consequences and solutions. PLoS One.

[3] Jarvis PR (2016). Improving emergency department patient flow. Clin Exp Emerg Med.

[4] Mistry B, Stewart De Ramirez S, Kelen G (2018). Accuracy and reliability of emergency department triage using the Emergency Severity Index: an international multicenter assessment. Ann Emerg Med.

[5] Farcas A, Ko J, Chan J, Malik S, Nono L, Chiampas G (2021). Use of incident command system for disaster preparedness: a model for an emergency department COVID-19 response. Disaster Med Public Health Prep.

[6] Hinson JS, Martinez DA, Schmitz PS (2018). Accuracy of emergency department triage using the Emergency Severity Index and independent predictors of under-triage and over-triage in Brazil: a retrospective cohort analysis. Int J Emerg Med.

[7] Rahmani F, Sepehri Majd P, Ebrahimi Bakhtavar H, Rahmani F (2018). Evaluating the accuracy of emergency nurses in correct triage using emergency severity index triage in Sina hospital of Tabriz: a cross-sectional analysis. J Emerg Pract Trauma.

[8] Ekins K, Morphet J (2015). The accuracy and consistency of rural, remote and outpost triage nurse decision making in one Western Australia Country Health Service Region. Australas Emerg Nurs J.

[9] Martin A, Davidson CL, Panik A, Buckenmyer C, Delpais P, Ortiz M (2014). An examination of ESI triage scoring accuracy in relationship to ED nursing attitudes and experience. J Emerg Nurs.

[10] Saban M, Dagan E, Drach-Zahavy A (2019). The relationship between mindfulness, triage accuracy, and patient satisfaction in the emergency department: a moderation-mediation model. J Emerg Nurs.

[11] Goldstein LN, Morrow LM, Sallie TA, Gathoo K, Alli K, Mothopeng TM, Samodien F (2017). The accuracy of nurse performance of the triage process in a tertiary hospital emergency department in Gauteng Province, South Africa. S Afr Med J.

[12] Öner-Şimşek D (2018). General overview of triage scales and determination of factors affecting emergency service applications in Turkey by logistic regression. J Soc Insur.

[13] Cetin SB, Eray O, Cebeci F, Coskun M, Gozkaya M (2020). Factors affecting the accuracy of nurse triage in tertiary care emergency departments. Turk J Emerg Med.

[14] Phukubye TA, Mbombi MO, Mothiba TM (2019). Knowledge and practices of triage amongst nurses working in the emergency departments of rural hospitals in Limpopo Province. Open Public Health J.

[15] Kerie S, Tilahun A, Mandesh A (2018). Triage skill and associated factors among emergency nurses in Addis Ababa, Ethiopia 2017: a cross-sectional study. BMC Res Notes.

[16] Ebrahimi M, Mirhaghi A, Mazlom R, Heydari A, Nassehi A, Jafari M (2016). The role descriptions of triage nurse in emergency department: a Delphi study. Scientifica (Cairo).

[17] Elgazzar SE (2021). Knowledge of triage and its correlated factors among emergency department nurses. Egypt J Health Care.

[18] Fathoni M, Sangchan H, Songwathana P (2010). Triage knowledge and skills among emergency nurses in East Java Province, Indonesia. Australas Emerg Nurs J.

[19] Afaya A, Azongo TB, Yakong VN (2017). Perceptions and knowledge on triage of nurses workingin emergency departments of hospitals in the Tamale Metropolis, Ghana. IOSR-JNHS.

[20] Aloyce R, Leshabari S, Brysiewicz P (2014). Assessment of knowledge and skills of triage amongst nurses working in the emergency centres in Dar es Salaam, Tanzania. Afr J Emerg Med.

[21] Asgari H, Omidi MR, Omidi N (2018). Evaluating the disaster triage knowledge of nurses personnel in public hospitals of Ilam. HDQ.

[22] Fathoni M, Sangchan H, Songwathana P (2013). Relationships between triage knowledge, training, working experiences and triage skills among emergency nurses in East Java, Indonesia. Nurse Media Journal of Nursing.

[23] AlShatarat M, Rayan A, Eshah NF, Baqeas MH, Jaber MJ, ALashtawy M (2022). Triage knowledge and practice and associated factors among emergency department nurses. SAGE Open Nurs.

[24] Murphy EJ, Tuot DS (2021). Assuring safety and efficacy of nurse triage for electronic consultation to improve access to specialty care. BMJ Qual Saf.

[25] Harmon S (2020). Same shift, different day—there are no VIPs in triage. Emerg Med News.

[26] Duko B, Geja E, Oltaye Z, Belayneh F, Kedir A, Gebire M (2019). Triage knowledge and skills among nurses in emergency units of Specialized Hospital in Hawassa, Ethiopia: cross sectional study. BMC Res Notes.

[27] Haghigh SH, Ashrafizadeh H, Mojaddami F, Kord B (2017). A survey on knowledge level of the nurses about hospital triage. J Nurs Educ.

[28] Reisi Z, Saberipour B, Adine M, Hemmatipour A, Shahvali EA (2018). The level of awareness of the emergency department nurses of the triage principles in teaching hospitals. J Nurs Midwifery Sci.

[29] Souza CC, Chianca TC, Cordeiro Júnior W, Rausch MD, Nascimento GF (2018). Reliability analysis of the Manchester Triage System: inter-observer and intra-observer agreement. Rev Lat Am Enfermagem.

[30] Steiner D, Renetseder F, Kutz A (2016). Performance of the Manchester Triage System in adult medical emergency patients: a prospective cohort study. J Emerg Med.

[31] McCann TV, Clark E, McConnachie S, Harvey I (2007). Deliberate self-harm: emergency department nurses' attitudes, triage and care intentions. J Clin Nurs.

[32] Andersson AK, Omberg M, Svedlund M (2006). Triage in the emergency department--a qualitative study of the factors which nurses consider when making decisions. Nurs Crit Care.

[33] Hicks FD, Merritt SL, Elstein AS (2003). Critical thinking and clinical decision making in critical care nursing: a pilot study. Heart Lung.

[34] Javadi S, Salimi T, Sareban MT, Dehghani MA (2016). Knowledge and practice of nurses regarding patients’ triage in emergency department. Iran J Emerg Med.

[35] Kalantarimeibidi M, Yadollahi A, Esfandiari S (2014). The effect of education on the knowledge and practice of emergency department’s nurses regarding the patients’ triage. Iran J Emerg Med.

[36] Forsgren S, Forsman B, Carlström ED (2009). Working with Manchester triage -- job satisfaction in nursing. Int Emerg Nurs.

[37] Salonen AH, Kaunonen M, Meretoja R, Tarkka MT (2007). Competence profiles of recently registered nurses working in intensive and emergency settings. J Nurs Manag.

[38] Chen SS, Chen JC, Ng CJ, Chen PL, Lee PH, Chang WY (2010). Factors that influence the accuracy of triage nurses' judgement in emergency departments. Emerg Med J.

[39] Jordi K, Grossmann F, Gaddis GM, Cignacco E, Denhaerynck K, Schwendimann R, Nickel CH (2015). Nurses' accuracy and self-perceived ability using the Emergency Severity Index triage tool: a cross-sectional study in four Swiss hospitals. Scand J Trauma Resusc Emerg Med.

[40] Bambi S, Ruggeri M (2017). [Triage duration times: a prospective descriptive study in a level 1° emergency department]. Prof Inferm.

[41] Villa S, Weber EJ, Polevoi S, Fee C, Maruoka A, Quon T (2018). Decreasing triage time: effects of implementing a step-wise ESI algorithm in an EHR. Int J Qual Health Care.

[42] Hamamoto J, Yamase H, Yamase Y (2016). Factors affecting the duration of nurses’ decision making in triage in Japan. Arch Emerg Med Crit Care.

[43] Dadashzadeh A, Abdolahzadeh F, Rahmani A, Ghojazadeh M (2014). Factors affecting triage decision-making from the viewpoints of emergency department staff in Tabriz hospitals. Iran J Crit Care Nurs.

[44] Madani F, Moosavi S, Jafaraghaee F, Leyli EK, Yazdanipour MA (2019). Factors associated with hospital triage decision making from the viewpoints of emergency nurses. J Adv Pharm Edu Res.

[45] Vargas JP, Hubloue I, Pinzón JJ, Duque AC (2019). The effect of training and experience on mass casualty incident triage performance: evidence from emergency personnel in a high complexity university hospital. Am J Disaster Med.

[46] Hammad K, Peng L, Anikeeva O, Arbon P, Du H, Li Y (2017). Emergency nurses' knowledge and experience with the triage process in Hunan Province, China. Int Emerg Nurs.

